# Loss of synaptic Munc13-1 underlies neurotransmission abnormalities in spinal muscular atrophy

**DOI:** 10.1007/s00018-025-05859-7

**Published:** 2025-08-29

**Authors:** Mehri Moradi, Chunchu Deng, Michael Sendtner

**Affiliations:** 1 Institute of Clinical Neurobiology, University Hospital Wuerzburg, Versbacher Str. 5, 97078 Wuerzburg, Germany; 2 Department of Rehabilitation Medicine, Tongji Hospital, Tongji Medical College, Huazhong University of Science and Technology, Jiefang Avenue, Wuhan, 430030 China

**Keywords:** Active zone, Axonal mRNA localization, Survival of motor neuron, Synapse degeneration, Munc13-2

## Abstract

Spinal muscular atrophy (SMA) is a devastating neurodegenerative disease characterized by degeneration of spinal motoneurons, leading to muscle atrophy and synaptic loss. SMN functions in mRNA splicing, transport, and local translation are crucial for maintaining synaptic integrity. Within the presynaptic membrane, the active zone orchestrates the docking and priming of synaptic vesicles. The Munc13 family proteins are key active zone components that operate precise neurotransmitter release in conjunction with voltage-gated Ca^2+^ channels (VGCCs). However, the role of Munc13s in synaptic dysfunction in SMA remains elusive. Our findings reveal that Munc13-1 loss, but not Munc13-2, is closely linked to synaptic aberrations in SMA. Specifically, *Munc13-1* mRNA localization in axons is dependent on Smn, and its disruption leads to impaired AZ assembly and VGCC clustering in motoneurons, ultimately reducing neuronal activity. In contrast, Munc13-2 does not appear to be essential for AZ assembly or motoneuron differentiation, as its functions can be compensated by Munc13-1. These findings highlight the pivotal role of Munc13-1 in synapse integrity and point to potential therapeutic targets for mitigating synaptic loss in SMA.

**Supplementary Information:**

The online version contains supplementary material available at 10.1007/s00018-025-05859-7.

## Introduction

Impaired synaptic function and degeneration are common pathological features of neurodegenerative diseases, including Spinal muscular atrophy (SMA) [1]. SMA is the second most common fatal autosomal recessive genetic disease with an incidence of 1 per 6,000 births [2]. SMA is caused by deletions of the Survival Motor Neuron 1 (*SMN1*) gene [2]. These mutations lead to degeneration of spinal motoneurons, loss of axons, denervation of neuromuscular junctions (NMJs), presynaptic accumulation of neurofilaments, endplate abnormalities, and muscle atrophy in SMA patients and animal models [3–5]. The SMN protein is required for the assembly of small nuclear ribonucleoproteins involved in pre-mRNA splicing [6] as well as regulation of axonal mRNA transport and local translation [7, 8]. Mouse models of SMA exhibit atrophic and smaller synapses, which are associated with impaired neurotransmitter release [9], disturbed clustering of voltage-gated Ca^2+^ channels (VGCCs) [10], and reduced evoked postsynaptic potential at neuromuscular endplates [11]. Motoneurons in SMA are believed to undergo degeneration in a “dying-back” manner, where the presynaptic terminal withdraws from the postsynaptic endplate, resulting in partially innervated endplates [12]. Additionally, the loss of axons in the ventral roots is observed to be more pronounced than the loss of motoneuron cell bodies, indicating a distal-to-proximal pattern of degeneration [12]. In SMA mice, NMJ defects manifest before the onset of clinical symptoms, suggesting that the NMJ represents an “early pathological target” in SMA [13].

Despite the emerging evidence about synaptic impairments in SMA, the cellular mechanisms underlying synapse dysfunction and degeneration are not well understood.

Neurotransmitter release at the presynaptic membrane is a finely tuned process initiated by Ca^2+^ entry through VGCCs [14]. This occurs at specialized regions within the presynaptic plasma membrane known as active zones (AZs) [15]. At the molecular level, AZs are densely populated with a complex array of proteins such as RIM, Munc13s, Piccolo (Pclo), and Bassoon (Bsn), which interact with synaptic vesicles (SVs) to orchestrate their docking, priming, and fusion with the plasma membrane [16]. At the AZ within the presynaptic membranes, the Munc13 family release factor proteins achieve the temporal and spatial precision of SV release events in coordination with VGCCs [14, 15]. Munc13-1 plays a crucial role in synaptic plasticity by regulating SV priming and modifying the fusion competence of the readily releasable pool [9, 17]. Munc13-1 KO mice exhibit severe neurological phenotypes including a complete failure of neurotransmitter release at central synapses [16] and NMJs [17], leading to paralysis and early postnatal death [18]. In humans, *UNC13A* mutations are associated with microcephaly, cortical hyperexcitability, and fatal myasthenia [19].

Thus, we asked whether alterations in Munc13s synaptic functions might be associated with pathomechanisms underlying neurotransmission and plasticity defects in SMA.

Here, we elucidated the roles of Munc13-1 and Munc13-2 in neurotransmission in a mouse model of SMA and revealed that loss of Munc13-1, but not Munc13-2, is associated with synaptic aberrations in SMA mice. Our findings indicate that axonal localization of Munc13-1 and *Munc13-2* mRNAs depends on Smn and is perturbed in SMA. Additionally, we show that loss of Munc13-1 leads to defective levels of AZ components and VGCCs in presynaptic membranes in motoneurons, contributing to impaired neuronal activity. Together, these data demonstrate a role for Munc13-1 in AZ assembly and neurotransmitter release in motoneurons, highlighting a potential mechanism for synaptic abnormalities in SMA.

## Materials and methods

### Animals

Mice were housed in the animal facility of the Institute for Clinical Neurobiology in compliance with national federal law and the guidelines of the Association for Assessment and Accreditation of Laboratory Animal Care. As SMA mouse model, we used the established C57Bl/6 N/Smn1tm1Hung Tg(SMN2)2Hung/J line with C57BL/6J background [20]. SMA litters (*Smn*^*−/−*^,*Hung*^*tg/+*^, or Smn KO), and control litters (*Smn*^*+/−*^,*Hung*^*tg/+*^ or control) were derived from cross-breeding of Smn^+/−^ to *Smn*^*−/−*^,*Hung*^*tg/tg*^. C57BL/6J mice were used as WT for control experiments. Munc13-1 KO mice (*Munc13-1*^*−/−*^*)*, and Munc13-2 KO mice *(Munc13-2*^*−/−*^) [16, 18] were obtained from Nils Brose from Goettingen, Germany and cross-bred in-house.

### Enrichment and culturing of primary mouse motoneurons

Primary mouse motoneurons were enriched via p75^NTR^ antibody panning and cultured as previously described [8, 21, 22]. For lentivirus transduction, cell suspensions were briefly exposed to lentiviral particles for 10 min at RT and then plated on pre-coated polyornithine and laminin211/221 (Biolamina, LN211-0501, and LN221-0501) dishes. This specific laminin isoform has been shown to promote axonal growth cone differentiation into presynaptic structures in cultured motoneurons [10, 23]. Cells were grown onto glass coverslips for immunofluorescence, 24-well plates for Western blot and qRT-PCR, and µ-dishes (Ibidi, 81156) for Ca^2+^ imaging. Motoneuron culturing into compartmentalized microfluidic chambers was conducted as described earlier [24].

### Immunocytochemistry

Cells were washed twice with pre-warmed PBS and fixed with 4% Paraformaldehyde (PFA) (ThermoFisher Scientific, 28908) for 10 min at RT and permeabilized with 0.1% Triton X-100. Block solution (2% BSA, 100 µg/ml saponin, and 0.25% sucrose in PBS) was added and incubated for 1 h at RT. Primary antibodies were diluted in block solution and incubated at 4 °C overnight. Primary antibodies were washed trice with TBST followed by secondary antibody incubation (diluted 1:500 in PBS) for 1 h at RT. Coverslips were embedded in Aqua Poly/Mount (Polysciences, 18606-20). For β-actin (Actβ) immunostaining, cells were permeabilized with ice-cold methanol for 5 min at −20 °C followed by a 5 min incubation at RT with 0.1% Triton X-100. Primary antibodies are as followed: rabbit polyclonal anti-Tau (Sigma-Aldrich, T6402, 1:1000), mouse monoclonal anti-α-Tubulin (Sigma-Aldrich, T5168, 1:1000), mouse monoclonal purified IgG anti-Basoon (Synaptic Systems, 141011, 1:500), guinea pig polyclonal antiserum anti-Piccolo (Synaptic systems, 142104, 1:500), rabbit polyclonal purified anti-RIM1/2 (Synaptic Systems, 140213, 1:500), rabbit polyclonal anti-Munc13-1 (Synaptic System, 126103, 1:500), rabbit polyclonal anti-Munc13-2 (Synaptic System, 126203, 1:400), rabbit polyclonal anti-Liprinα1 (Merck Millipore, ABT268, 1:500), guinea pig polyclonal purified anti-Ca^2+^ channel N-type alpha-1B (Ca_v_2.2) (Synaptic System, 152305, 1:250), mouse monoclonal anti-SMN (BD Biosciences, 610647, 1:5000), and mouse monoclonal anti-β-actin (GeneTex, GTX26276, 1:1000). Secondary antibodies are as followed: donkey anti-mouse IgG (H + L) (Alexa Fluor 488, Jackson ImmunoResearch, 715-545-150), donkey anti-rabbit IgG (H + L) AffiniPure (Alexa Fluor 488, Jackson ImmunoResearch, 711-545-152), donkey anti-rabbit IgG (H + L) AffiniPure (Cy3, Jackson ImmunoResearch, 711-165-152), and donkey anti-guinea pig IgG (H + L) AffiniPure (Cy5, Jackson ImmunoResearch, 706-175-148).

### Single-molecule fluorescence in situ hybridization (smFISH)

smFISH was conducted following the manufacturer’s instructions (ThermoFisher Scientific), as described earlier [8]. Briefly, after 10 min fixation at RT with paraformaldehyde lysine phosphate (PLP) buffer (4% PFA, 5.4% glucose, and 10 mM sodium metaperiodate, pH 7.4), cells were permeabilized for 4 min at RT with a supplied detergent solution. Cells were treated with proteinase K (diluted 1:8000 in PBS) for 4 min at RT. Hybridization probes (diluted 1:100 in hybridization buffer) were incubated at 40 °C overnight. Preamplifier, amplifier, and label probe oligonucleotides (diluted 1:25 in respective amplification buffers) were incubated each for 1 h at 40 °C. Following the washing steps, cells were immunostained for Tau to visualize the neurite boundaries.

### Immunohistochemistry

For immunohistochemistry, TVA muscles were dissected from P5 Smn KO mice, and diaphragm tissues were dissected from P0 Munc13-1 KO and P5 Munc13-2 KO mice. Dissected muscle preparations were transferred into an extracellular physiological solution (135 mM NaCl, 12 mM NaHCO3, 5 mM KCl, 1 mM MgCl_2_, 2 mM CaCl_2_, 20 mM glucose) and fixed with 4% PFA at 4 °C for 90 min. PFA was quenched by 30 min incubation with 0.1 M glycine followed by permeabilization steps with 1% Triton X-100 (twice for 5 min, twice for 10 min, and twice for 30 min). Block solution (5% BSA, 0.1% Triton X-100 in PBS) was added and incubated at RT for 3 h. Primary antibodies diluted in block solution were incubated for two nights at 4 °C. Following wash steps with 0.1% Triton X-100 in PBS at RT, secondary antibodies were added together with α-Bungarotoxin (ThermoFisher Scientific, B13422, 1:1000) and incubated at RT for 1 h. Preparations were washed with 0.1% Triton X-100 in PBS, rinsed briefly in water, and embedded with Aqua-Poly/Mount. Alexa Fluor 488-conjugated α-Bungarotoxin was used to label postsynaptic membranes (AChRs) in NMJs. All the incubation steps were performed on a shaker. Following primary and secondary antibodies were used: guinea pig polyclonal anti-Synaptophysin1 (Synaptic Systems, 101004, 1:1000), rabbit polyclonal anti-Ca^2+^ channel P/Q-type (Ca_v_2.1) (Synaptic Systems, 152203, 1:500), rabbit polyclonal anti-Munc13-1 (Synaptic System, 126103, 1:400), rabbit polyclonal anti-Munc13-2 (Synaptic System, 126203, 1:400), guinea pig polyclonal antiserum anti-Munc13-1 (Synaptic Systems, 126104, 1:500), donkey anti-rabbit IgG (H + L) AffiniPure (Cy3, Jackson ImmunoResearch, 711-165-152, 1:500), donkey anti-guinea pig IgG (H + L) AffiniPure (Cy5, Jackson ImmunoResearch, 706-175-148, 1:500).

### Ca^2+^ imaging and data quantification

For Ca^2+^ imaging with cultured motoneurons, calcium indicator Oregon Green™ 488 BAPTA-1, AM, cell-permeant (ThermoFisher Scientific, O6807) was used. The calcium indicator was dissolved in Pluronic F-127/DMSO and sonicated in an ultrasonic bath for 2 min to prepare a 5 mM stock solution. Following twice washing steps with pre-warmed Ca^2+^ imaging buffer (135 mM NaCl, 6 mM KCl, 1 mM MgCl_2_, 1 mM CaCl_2_, 10 mM HEPES, and 5.5 mM glucose), Ca^2+^ indicator (5 µM diluted in Ca^2+^ imaging buffer) was added into cultured motoneuron dishes and incubated with for 15 min at 37 °C in a CO_2_ incubator. Residual calcium indicator dye was removed by twice washing with Ca^2+^ imaging buffer and cells were imaged in 2 ml Ca^2+^ imaging buffer supplemented with 3.5 ng/ml BDNF. A Nikon inverted epifluorescence microscope (TE2000) was used for time-lapse imaging. This was equipped with a 60 × 1.4-NA objective, a perfect focus system, an Orca Flash 4.0 V2 camera (Hamamatsu Photonics), an LED fluorescence light for excitation at 470 nm, and Nikon Element image software. Cells were imaged at 37 °C with 5% CO_2_ using a TOKAI HIT CO, LTD heated stage chamber. To monitor the spontaneous Ca^2+^ spikes, time-lapse images were taken at 500 ms intervals for 7 min. For membrane depolarization with KCl, cells were first imaged at 500 ms intervals for 1 min and then received 10 µl of 90 mM KCl, followed by another minute of imaging. Images were taken at 16-bit with a resolution of 1.024 × 1.024-pixel and a 2 × 2 binning. Quantifications of Ca^2+^ spikes were conducted in regions of interest (ROIs) within growth cones using Fiji. For this, intensity values were first measured from all time-lapse frames using the Fiji plug-in “dynamic Z-axis profile”. The measured average intensities were normalized to the average intensities of the first 10 frames before a spontaneous Ca^2+^ spike appeared (F_0_) and plotted (F/F_0_). For KCl pulse experiments, the measured time-lapse intensities were normalized to the average of the first 20 frames immediately before KCl application (F_0_) and plotted (F/F_0_). BAR Plugin of Fiji was used for counting the Ca^2+^ spikes.

### RNA extraction and quantitative RT-PCR (qRT-PCR)

For RNA extraction from total cell lysates, spinal cord, and brain tissues, NucleoSpin RNA purification kit (MACHEREY-NAGEL, 740955.50) was used. RevertAid First Strand cDNA Synthesis Kit (ThermoFisher Scientific, K1621) was used for reverse transcription with random primers. RNA extraction from microfluidic chambers was applied as previously described [8]. qRT-PCR was performed on a LightCycler 1.5 thermal cycler (Roche) using Luminaris HiGreen qPCR Master Mix (ThermoFisher Scientific, K0992). The relative expression was measured according to the ΔΔCt method using Gapdh for data normalization.

Following primers were used for qRT-PCR: Gapdh (forward) 5’-AACTCCCACTCTTCCACCTTC-3’ and (reverse) 5’-GGTCCAGGGTTTCTTACTCCTT-3’, Munc13-1 (forward) 5’-CACCACGCCCACCTACTGCTA-3’ and (reverse) 5’-TTGCGCTCGCGGATCT-3′, Munc13-2 (forward) 5’-CTTGGCAGATGATAATGAGTA-3’ and (reverse) 5’-GGTAGTCACTGTCTCGGTC-3′, Liprinα1 (forward) 5’-GATGGACTGCTTGACGGAAAC-3’ and (reverse) 5’-GGCCATTGCTTCACGGAC-3′, Bsn (forward) 5’-GCTGCCAGCCAACCAG-3’ and (reverse) 5’-CCACCAGGGAGGATCTTAGAG-3’, Pclo (forward) 5’-CCCGACCCATCCAAGGATATG-3’ and (reverse) 5’-TGGTTGAATGCGGAGTTGCT-3’, and RIM2 (forward) 5’-CAGACCCTGGCTACTCCTGC-3’ and (reverse) 5’-TACGGTGCTGGCAGTGTCTTG.

### Western blotting

For Western blotting of primary mouse motoneurons, 300,000 cells were plated and grown for 7 days. Cells were lysed directly in 1 × Laemmli buffer (125 mM Tris, pH 6.8, 10% SDS, 50% glycerol, 25% β-mercaptoethanol, and 0.2% bromophenol blue). Lysates were boiled at 99 °C for 5 min, briefly centrifuged, and loaded onto 4–12% gradient SDS-PAGE gels. PVDF membranes were applied for blotting. For Western blot with brain and spinal cord, tissues were lysed in RIPA buffer (10 mM Tris-HCl, pH 8.0, 1 mM EDTA, 0.5 mM EGTA, 1% Triton X-100, 0.1% Sodium Deoxycholate, 0.1% SDS, 140 mM NaCl), protein concentration was measured using Pierce BCA Protein Assay Kit (ThermoFisher Scientific, A55860), and 20 µg total protein was loaded. For Western blot with crude synaptosome fractions, cortices were dissected from P0 Munc13-1 KO and P5 Munc13-2 KO mice and synaptosome fractions were prepared as previously described (55). Briefly, cortices were homogenized in 500 µl cold sucrose lysis buffer (0.32 M sucrose, 5 mM HEPES, 1 × protease inhibitor cocktail (Roche)) and centrifuged at 1000 × g for 10 min at 4 °C. Pellets (P1) were discarded and supernatants (S1) were centrifuged at 12,000 × g for 20 min at 4 °C. Resulting supernatants (S2) were discarded and pellets (P2) containing crude synaptosomes were resuspended in 100 µl PBS. Protein concentration was measured and 20 µg total protein was loaded onto 4–12% SDS gels. Primary antibodies were incubated overnight at 4 °C, and secondary antibodies were incubated for 1 h at RT. ECL reagents (GE Healthcare) were used for the membrane developing. The following antibodies were used: rabbit polyclonal anti-Calnexin (Enzo Life Sciences, ADI-SPA-860-F, 1:6000), rabbit polyclonal anti-Munc13-1 (Synaptic Systems, 126103, 1:5000), rabbit polyclonal anti-Munc13-2 (Synaptic Systems, 126203, 1:5000), mouse monoclonal anti-α-Tubulin (Sigma-Aldrich, T5168, 1:5000), mouse monoclonal anti-β-actin (GeneTex, GTX26276, 1:5000), mouse monoclonal anti-SMN (BD Biosciences, 610647, 1:5000), guinea pig polyclonal anti-Synaptophysin1 (Synaptic Systems, 101004, 1:5000), peroxidase AffiniPure donkey anti-goat IgG (H + L) (Biozol, 705-035-003, 1:10000), peroxidase AffiniPure donkey anti-mouse IgG (H + L) (Biozol, 715-035-151, 1:10000), peroxidase AffiniPure goat anti-guinea pig IgG (H + L) (Jackson, 106-035-003, 1:10000), and peroxidase AffiniPure goat anti-rabbit IgG (H + L) (Biozol, 111-035-144, 1:10000).

### Plasmid cloning of shRNA constructs targeting Munc13s and lentivirus production

For cloning of Munc13-1 overexpression lentivirus construct, a plasmid harboring the coding region (cDNA) of endogenous mouse Munc13-1 was purchased from GenScript and inserted into a lentivirus backbone vector with the Ubiquitin promotor using NEBuilder^®^ HiFi DNA Assembly Cloning Kit (New England Biolabs, E5520S). The pSIH-H1 vectors with shRNA-targeting mouse Munc13-1 and Munc13-2 were generated as previously described [8]. The sequences of the antisense oligos used for shRNA cloning are as follows; Munc13-1: 5’-TCCCGTGTGAAACAAAGGT-3’, and Munc13-2: 5’-CGGAATAAACCAGAGATCT-3’. Lentiviruses were produced in HEK^293T^ cells using TransIT-293 (Mirus, MIR2706) for transfection [25, 26]. pCMV-VSVG and pCMVΔR8.91 were employed as helper plasmids for the lentivirus production, and viral supernatants were collected by ultracentrifugation 60–72 h after transfection. The virus titer was assessed in NSC^34^ cells using serial dilutions.

### Image acquisition and processing

Images were acquired with an Olympus Fluoview 1000 confocal microscope equipped with a 60 × 1.35-NA oil objective. For cultured motoneurons, 16-bit images with a resolution of 800 × 800 pixels were taken from single z-stacks. For neuromuscular junctions (NMJs), confocal imaging involved 6 z-stacks at 0.5 μm intervals, with maximum projection images presented for illustration. Super-resolution SIM imaging was performed with an ELYRA S.1 SIM, using a Plan-Apochromat 63× NA 1.4 oil objective and excitation lasers with wavelengths of 405 nm (50 mW), 488 nm (100 mW), 561 nm (100 mW), and 642 nm (150 mW). Laser power was adjusted between 2 and 5% with an integration time of 200 ms. For SIM, z-stacks of 110 nm intervals were captured, and maximum projection images were used for representation. The 16-bit raw images were processed with Zeiss ZEN 3.0 SR FP2 black software to reconstruct super-resolution images. Channel alignment was performed using fiducial markers (ThermoFisher Scientific, TetraSpeck™ Microspheres, 0.2 μm, fluorescent blue/green/orange/dark red, T7280). Fiji was used for image processing and analysis. Linear contrast enhancement was implemented to all representative images using Adobe Photoshop 24.2.0 for improved visibility.

### Data analysis

To quantify immunofluorescence signals in growth cones and somata, mean gray values from unprocessed raw images were measured using Fiji after background subtraction. For assessing immunofluorescence signals at NMJs, average projections from multiple z-stack images were first created, and mean gray values were measured within the SynPhy-positive regions. All intensity measurements were normalized to the average intensity of the control group from the same experiment. For NMJ colocalization analysis, average z-stack projections were generated using Fiji, with NMJs defined as the region of interest, and Pearson R-values were calculated using Fiji. For the colocalization analysis of SIM data, single optical sections of 16-bit raw images were used to compute the Pearson R-value with Fiji, defining the entire growth cone as the region of interest. All immunostaining experiments were performed and analyzed in a blinded manner.

### Statistical analysis

For creating graphs and performing statistical analyses GraphPad Prism 10 was utilized. Data are presented as bar graphs or scatter dot plots, with error bars indicating mean ± SEM, or as violin plots, with the median shown as dashed lines. Individual data points are shown as symbols, always when *n* ≤ 10. Statistical significance between two groups was assessed using the Mann-Whitney U test. For all experiments, the “n” number refers to the total number of analyzed cells, and the “N” number refers to the number of independent biological replicates. In most experiments, data were obtained from cultures prepared from at least *N* = 3 independent mice, with each mouse contributing one culture. Therefore, in most experiments, the total number of cultures used for analysis was *N* = 3, derived from *N* = 3 independent biological samples. Statistical analyses were performed based on these independent biological replicates rather than individual observations or technical replicates within the same experiment. For Ca^2+^ imaging experiments, statistical analysis were performed on pooled data from all four independent experiments.

## Results

### Transcripts for *Munc13s*, and *Liprinα1*, but not *Pclo*, *Bsn*, and *RIM* are enriched in motor axons

RNA sequencing studies have shown that transcriptomes of neuronal processes, dendrites [27, 28], and axons [24, 29, 30] include transcripts encoding synapse-related proteins. The Smn protein plays a key role in axonal mRNA transport and local translation of various targets [7, 8], and the disruption of these functions is linked to impaired synaptic transmission in SMA [9, 10]. This raises the possibility that improper mRNA localization of AZ components may contribute to synapse dysfunction in SMA.

**Fig. 1:**
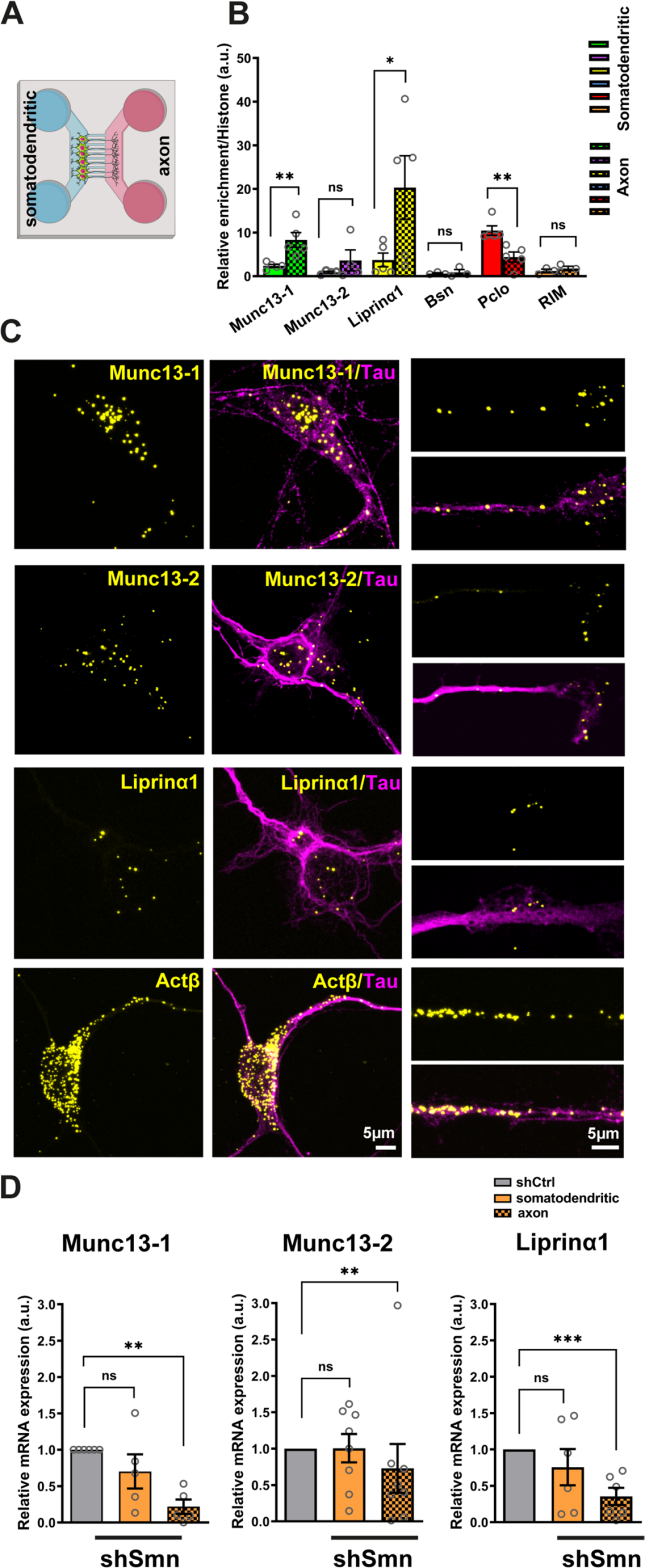
Transcripts for active zone proteins are enriched in distal axons of cultured mouse motoneurons.**A** Schematic representation of implied microfluidic chambers. **B** qRT-PCR was used to assess the relative enrichment of mRNAs encoding AZ proteins in axonal versus somatodendritic compartments of WT motoneurons, using *Histone H0* mRNA as a reference. mRNAs for *Munc13-1* (**P = 0.004), and *Liprinα1* (*P = 0.0476) are significantly enriched in axons of cultured WT motoneurons compared to mRNAs for *Histone H0*. In contrast, transcripts of *Pclo* (**P = 0.004) are enriched in the somatodendritic compartment of cultured motoneurons (*N* = 3–5 independent cultures/mice). **C** Representative images of smFISH with cultured motoneurons showing axonal localization of transcripts of *Munc13-1*, *Munc13-2*, and *Liprinα1*, in cultured motoneurons. **D** qRT-PCR was employed to assess the mRNA levels of AZ proteins in axonal and somatodendritic compartments of control and Smn knockdown motoneurons. In Smn knockdown motoneurons, the mRNA levels of *Munc13-1* (**P = 0.0022, *N* = 5 independent cultures/mice), Munc13-2 (**P = 0.0016, *N* = 8 cultures/mice), and Liprinα1 (***P = 0.0006, *N* = 6 independent cultures/mice) were significantly reduced in axons, but not in the somatodendritic compartment. In **B** and **D**, data are presented as mean ± SEM. *P ≤ 0.05 (One-tailed Mann-Whitney U test)

To explore this hypothesis, we investigated the localization of transcripts for AZ proteins within cultured mouse motoneurons using compartmentalized microfluidic chambers (Fig. 1A). Quantitative real-time PCR (qRT-PCR) analysis revealed a significant enrichment of transcripts for the AZ proteins including *Munc13-1*, and *Liprinα1* in distal axons compared to the somatodendritic compartment (Fig. 1B). Although *Munc13-2* transcripts also showed enrichment in axons, this was not statistically significant (Fig. 1B). In contrast, transcripts for *Pclo* were significantly enriched in the somatodendritic compartment (Fig. 1B). To further validate these findings, we employed single-molecule fluorescence in situ hybridization (smFISH) to visualize the subcellular localization of these transcripts. The smFISH results confirmed the localization of *Munc13-1*, *Munc13-2*, and *Liprinα1* transcripts in distal axons in cultured motoneurons (Fig. 1C).

### The presynaptic active zone is impaired in Smn KO motoneurons

Next, we assessed the impact of Smn loss of function on the axonal localization of transcripts for AZ proteins in cultured spinal motoneurons. To this end we employed a previously described and validated shRNA lentiviral construct to specifically target the mouse Smn [24]. Following Smn knockdown, qRT-PCR analysis demonstrated a significant reduction in the levels of *Munc13-1*, *Munc13-2*, and *Liprinα1* transcripts in axons of cultured motoneurons (Fig. 1D). These data suggest that Smn is crucial for the proper mRNA localization of these AZ components in motor axons. Since Smn is also involved in mRNA splicing, we investigated whether the loss of Smn affects the overall expression of AZ proteins. qRT-PCR analysis revealed a slight but statistically significant reduction in the total expression of AZ components in the brains of Smn KO mice, while no significant changes were observed in the spinal cord (Fig. S1A). The absence of significant differences in the spinal cord may be attributed to cellular heterogeneity, as the presence of various cell types, including motoneurons, interneurons, and glia, could dilute motoneuron-specific changes. These slight alterations in the brain, however, could be attributed to the severe synaptic loss observed in the late stages of SMA using P10 Smn KO mice. To evaluate this possibility, we analyzed the total expression of Munc13 isoforms in cultured motoneurons from E12.5 Smn KO mice using qRT-PCR. The results showed no significant reduction in *Munc13-1* and *Munc13-2* mRNA levels in Smn KO motoneurons at embryonic stages (Fig. S1B).

**Fig. 2:**
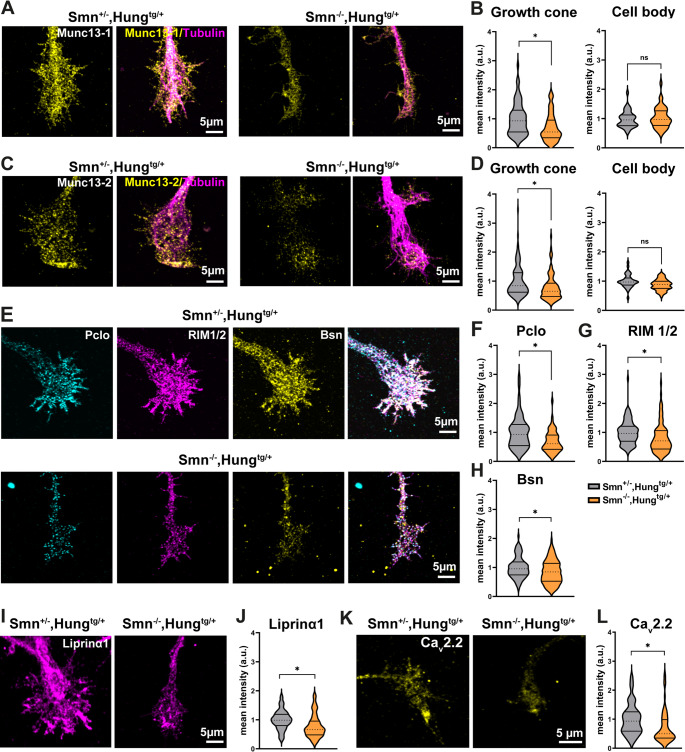
Cultured Smn KO motoneurons exhibit reduced levels of active zone components.**A**, **C**, **E**, **I**, and **K** Representative images of axonal growth cones of cultured motoneurons from Smn^+/−^,Hung^tg/+^ and Smn^−/−^,Hung^tg/+^ mice immunostained against active zone proteins. **B** and **D** Graphs show reduced Munc13-1 (**B**: *P = 0.0143, *n* = 144–162 cells, *N* = 4 independent cultures/mice for each genotype), and Munc13-2 (**D**: *P = 0.0143, *n* = 106–110 cells, *N* = 4 independent cultures/mice for each genotype) protein levels in axonal growth cones but not cell bodies of Smn KO motoneurons. **F**-**H** Graphs indicate significant reduction in protein levels of Pclo (**F**: *P = 0.05, *n* = 55–64 cells), RIM1/2 (**G**: *P = 0.0143, *n* = 96–99 cells), Bsn (**H**: *P = 0.0143, *n* = 101–122 cells), Liprinα1 (**J**: *P = 0.0286, *n* = 74–88 cells), and Ca_v_2.2 (**L**: *P = 0.0143, *n* = 70 cells) in axonal growth cones of cultured Smn KO motoneurons (*N* = 3–4 independent cultures/mice for each genotype). In **B**, **D**, **F**-**H**, **J** and **L**, data are presented as violin plots with the median shown as dashed lines. *P ≤ 0.05 (One-tailed Mann-Whitney U test)

We then examined the distribution and levels of key AZ proteins in cultured motoneurons derived from Smn KO mice. Immunostaining revealed a marked reduction in Munc13-1 and Munc13-2 protein levels in Smn KO motoneurons (Fig. 2A-D). Importantly, these reductions were observed specifically in axonal growth cones, with no significant changes detected in cell bodies (Fig. 2B, D). To confirm these findings, we conducted Western blot assays on total lysates from cultured Smn KO motoneurons, as well as whole-tissue brain and spinal cord lysates from Smn KO and control mice. The results showed a minor reduction in Munc13-1 protein levels in cultured Smn KO motoneurons (Fig. S1C), but no significant changes in its levels in brain and spinal cord tissues (Fig. S1D, E).

In contrast, Munc13-2 protein levels were elevated in total lysates from cultured Smn KO motoneurons (Fig. S1F), as well as in brain (Fig. S1G) and spinal cord tissues (Fig. S1H) of Smn KO mice, suggesting a compensatory upregulation mechanism. To validate the specific reduction of Munc13-1 protein levels in the axonal growth cones of cultured Smn KO motoneurons, we transduced these neurons with a previously described and validated lentiviral construct expressing human SMN fused to GFP (GFP-SMN) [31]. Importantly, overexpression of this construct restored Munc13-1 protein levels in the axonal growth cones of Smn KO motoneurons (Fig. S1I, J).

**Fig. 3:**
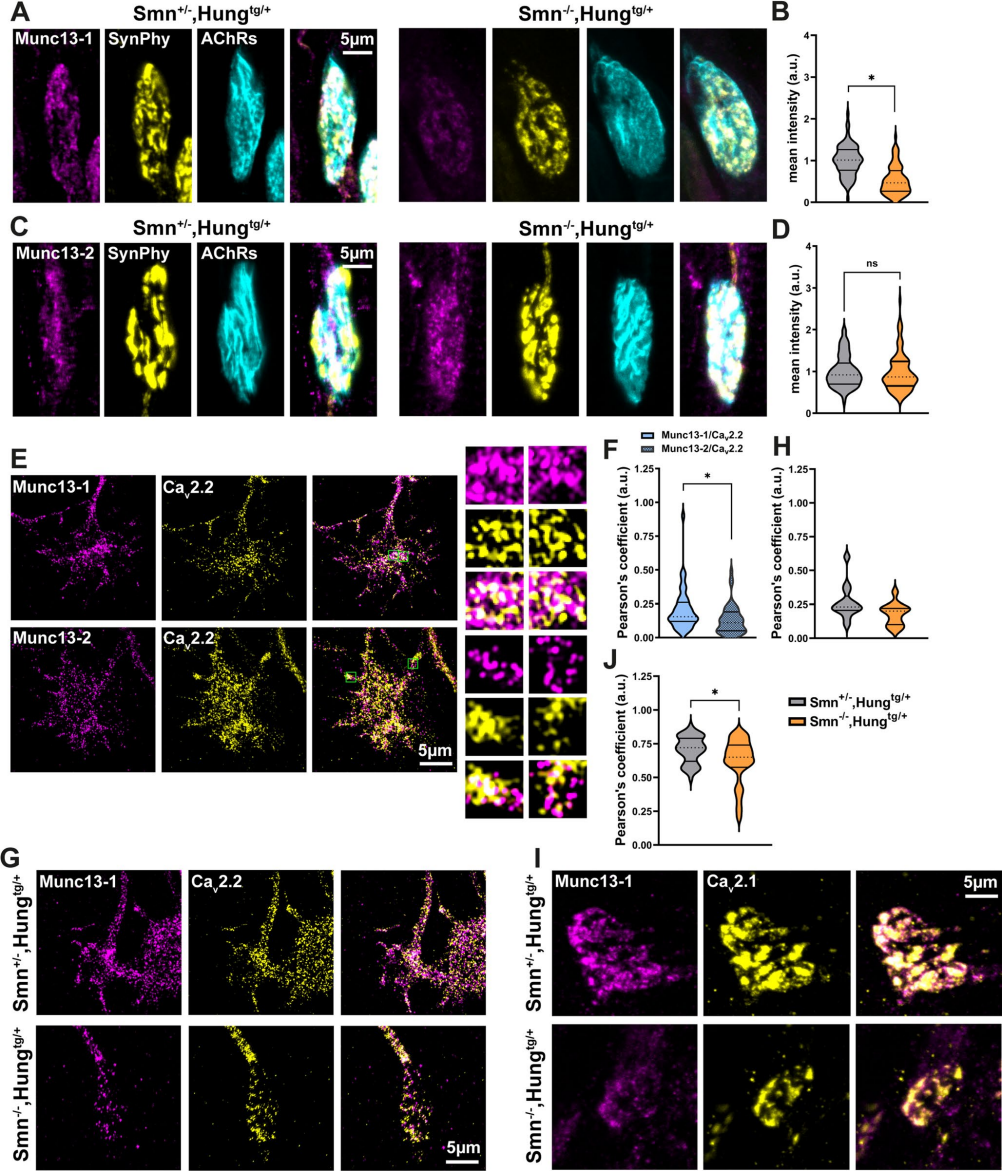
The spatial colocalization between Munc13-1 and VGCCs is altered at NMJs from Smn KO mice.**A** and **C** Representative images of NMJs from TVA muscles isolated from P5 control and Smn KO mice. **B** and **D** Munc13-1 (**B**: *P = 0.05, *n* = 79–86 NMJs, *N* = 3 independent experiments), but not Munc13-2 (**D**: *n* = 80–94 NMJs, *N* = 3 independent experiments) levels are reduced at NMJs in Smn KO mice. **E** Representative SIM images of axonal growth cones of cultured motoneurons stained against Munc13s and Ca_v_2.2. Inset on the right side: Zoom-in indicates colocalization between Munc13 isoforms and Ca_v_2.2. **F** Colocalization analysis of SIM data, as represented in panel **E**, reveals that in axonal growth cones of cultured WT motoneurons, the colocalization between Munc13-1 and Ca_v_2.2 is significantly higher than the colocalization between Munc13-2 and Ca_v_2.2 (*P = 0.05, *n* = 44–49 cells, *N* = 3 independent cultures/mice). **G** Representative SIM images of axonal growth cones of control and Smn KO motoneurons indicating Munc13-1 and Ca_v_2.2 colocalization. **H** Quantification of data represented in **G** demonstrates reduced colocalization between Munc13-1 and Ca_v_2.2 at axonal growth cones in Smn KO motoneurons compared to control (*n* = 13 cells, *N* = 2 independent cultures/mice for each genotype). **I** Representative images of NMJs from TVA muscles from P5 control and Smn KO mice. **J** Quantification of data represented in **I** shows reduced colocalization between Munc13-1 and Ca_v_2.1 at NMJs in Smn KO mice (*P = 0.05, *n* = 50–65 NMJs, *N* = 3 independent experiments). Data are presented as violin plots with the median shown as dashed lines. *P ≤ 0.05 (One-tailed Mann-Whitney U test)

Further analysis of other AZ components including Pclo, RIM1/2, Bsn, Liprinα1, and Ca_v_2.2 revealed a broader disruption in AZ protein levels within the growth cones of Smn KO motoneurons (Fig. 2E-L). Additionally, we assessed the levels of Munc13 isoforms at NMJs from the transverse abdominis muscles (TVA) of Smn KO mice. Notably, we observed a significant reduction in Munc13-1 levels at NMJs, while Munc13-2 levels remained unaltered (Fig. 3A-D). The specificity of the Munc13-1 and Munc13-2 antibodies was validated by Western blot analysis of the crude synaptosome fractions from the cortices of Munc13s KO mice (Fig. S2A, B), as well as by immunohistochemistry analysis of NMJs from Munc13s KO mice (Fig. S2C, D).

These findings suggest that loss of Smn severely reduces the levels of AZ components within the presynaptic membranes, in particular Munc13-1. This disruption likely contributes to the synaptic abnormalities seen in SMA, underscoring the vulnerability of the synaptic assembly process in the absence of a functional Smn protein.

### Munc13-1 tethers VGCCs into presynaptic AZs in motoneurons

At presynaptic AZs, Munc13-1 interacts with proteins like RIM and RIM binding protein (RIM-BP), forming a complex that helps tether SVs close to VGCCs [16, 17]. This precise spatial alignment between Munc13s and VGCCs ensures temporal and spatial regulation of neurotransmitter release [18, 19]. To gain a detailed view of the spatial colocalization between Munc13 isoforms and Ca_v_2.2 (a subtype of VGCCs) in axonal growth cones of wildtype (WT) motoneurons, we employed Structured Illumination Microscopy (SIM) (Fig. 3E). Quantitative colocalization analysis revealed that in axonal growth cones of WT motoneurons, the colocalization between Munc13-1 and VGCCs (Ca_v_2.2) is significantly higher than the colocalization between Munc13-2 and VGCCs (Fig. 3F). This finding is consistent with previous studies that demonstrated a tighter coupling between Unc13a and VGCCs at AZs in *Drosophila* NMJs [14]. Intriguingly, SIM imaging revealed that the colocalization between Munc13-1 and Ca_v_2.2 appears to be reduced at axonal growth cones in cultured Smn KO motoneurons (Fig. 3G, H). This reduction indicates a disruption in the spatial organization of these critical synaptic components. Similarly, confocal imaging of NMJs from the TVA muscles of Smn KO and control mice showed a significant decrease in the colocalization between Munc13-1 and Ca_v_2.1 (another VGCC subtype) at NMJs in Smn KO mice (Fig. 3I, J). These findings suggest that the loss of Smn disrupts the spatial organization of Munc13-1 and VGCCs at both axonal growth cones and NMJs, potentially contributing to the synaptic dysfunction in SMA.

### Depletion of Munc13-1 reduces the levels of AZ components and diminishes neuronal activity

**Fig. 4:**
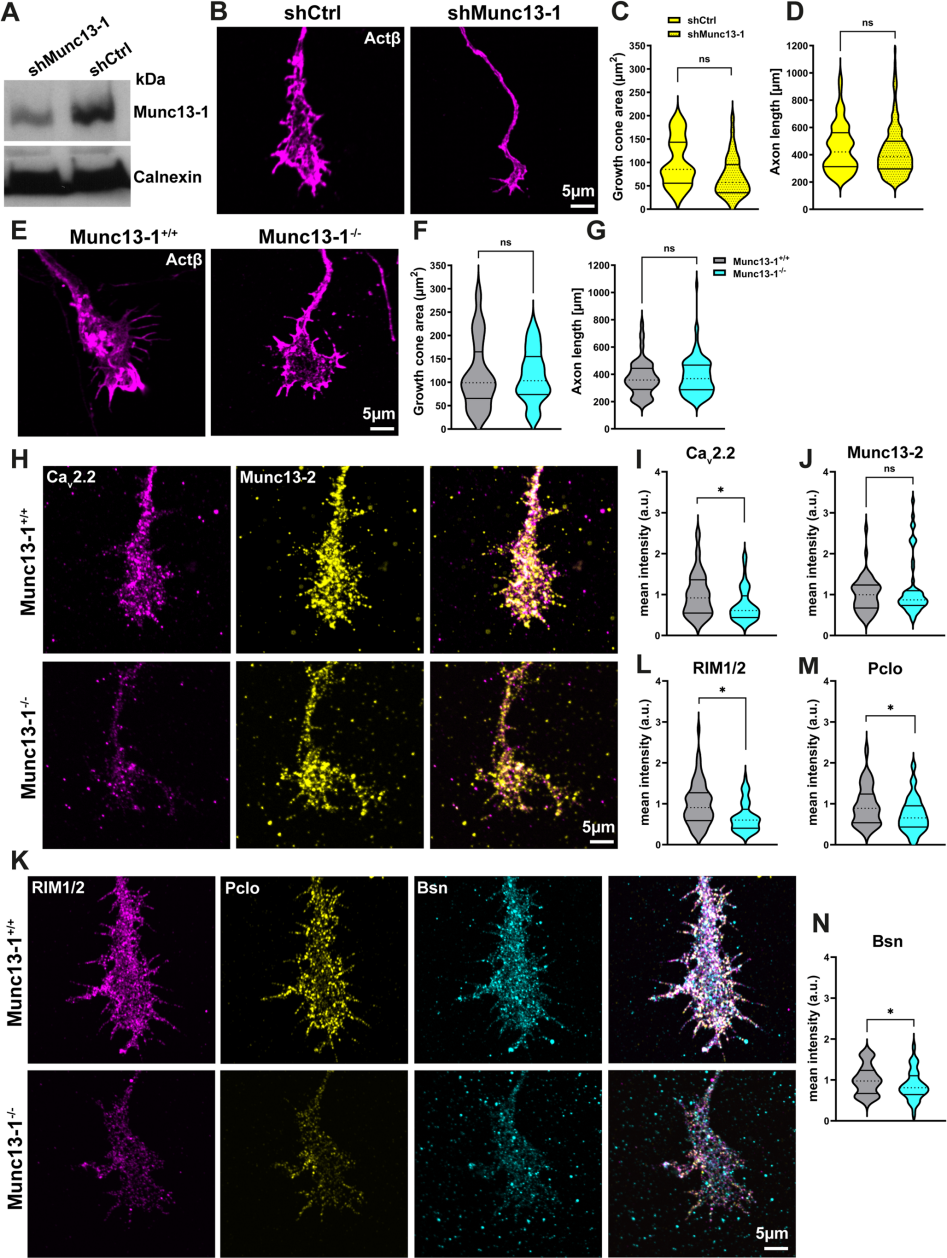
Munc13-1 loss of function reduces the levels of AZ components and impairs synapse differentiation in motoneurons.**A** Western blot with total lysates from cultured motoneurons transduced with lentiviruses of shCtrl and shRNA targeting Munc13-1. **B** Representative images of axonal growth cones of cultured motoneurons transduced with shCtrl or shMunc13-1 lentiviruses, stained against β-actin. **C** Munc13-1-depleted motoneurons appear to exhibit smaller axonal growth cones compared to control, although not significant (*n* = 43–45 cells, *N* = 3 independent cultures/mice). **D** shRNA-mediated Knockdown of Munc13-1 does not affect axon outgrowth (*n* = 205–215 cells, *N* = 3 independent cultures/mice). **E** Representative images of axonal growth cones of control and Munc13-1 KO motoneurons stained against β-actin. **F** and **G** The area of axonal growth cones (*n* = 35–38 cells), as well as the axon length (*n* = 98–105 cells), are comparable between control and Munc13-1 KO motoneurons (*N* = 3 independent cultures/mice for each genotype). **H** and **K** Representative images of axonal growth cones of cultured control and Munc13-1 KO motoneurons stained against AZ proteins and Ca_v_2.2. **I** and **J** The immunoreactivity of Ca_v_2.2 (*P = 0.05, *n* = 50–52 cells), but not Munc13-2 (*n* = 52–54 cells, *N* = 3 independent cultures/mice for each genotype), is decreased in axonal growth cones of Munc13-1 KO motoneurons. **L**-**N** Graphs demonstrate reduced immunoreactivity of RIM1/2 (**L**: *P = 0.05, *n* = 64–66 cells), Pclo (**M**: (*P = 0.05, *n* = 60–65 cells), and Bsn (**N**: (*P = 0.05, *n* = 64–66 cells) in axonal growth cones of Munc13-1 KO motoneurons (*N* = 3 independent cultures/mice for each genotype). Data are presented as violin plots with the median shown as dashed lines. *P ≤ 0.05 (One-tailed Mann-Whitney U test)

We investigated the role of Munc13-1 and Munc13-2 in motoneuron development and function by employing shRNA constructs to selectively knockdown these proteins. Additionally, we analyzed the phenotypes of cultured motoneurons obtained from Munc13s KO mice. As shown in Fig. 4A, Western blot analysis confirmed effective knockdown of Munc13-1 in motoneurons transduced with shRNA targeting Munc13-1. Imaging of axonal growth cones revealed that Munc13-1-depleted motoneurons exhibit smaller growth cones compared to the control, although the difference was not statistically significant (Fig. 4B, C). This suggests that Munc13-1 depletion might impair axonal growth cone differentiation. Despite this reduction in growth cone size, axon outgrowth was unaffected by Munc13-1 depletion (Fig. 4D). Interestingly, measurements of growth cone area and axon length in Munc13-1 KO motoneurons were comparable to those in control motoneurons (Fig. 4E-G). This might be due to compensatory mechanisms activated in Munc13-1 KO motoneurons. Furthermore, to elucidate whether Munc13-1 loss recapitulates SMA phenotype, we examined the AZ components and VGCCs in Munc13-1 KO motoneurons by immunostaining (Fig. 4H, K). These quantitative analyses showed that Ca_v_2.2 levels were reduced in axonal growth cones of Munc13-1 KO motoneurons (Fig. 4H, I), while Munc13-2 levels remained unchanged (Fig. 4H, J). In line with this, the levels of RIM1/2, Pclo, and Bsn were reduced in axonal growth cones of Munc13-1 KO motoneurons (Fig. 4K-N). We also assessed the Smn protein levels in Munc13-1 knockdown motoneurons. Western blot analysis of total lysates revealed no significant difference in overall Smn levels between control and Munc13-1 knockdown motoneurons (Fig. S3A, B). Additionally, immunocytochemistry revealed that Smn levels in axonal growth cones remained unchanged following Munc13-1 depletion (Fig. S3C, D). These findings indicate that Munc13-1 is not involved in the regulation of Smn expression.

**Fig. 5:**
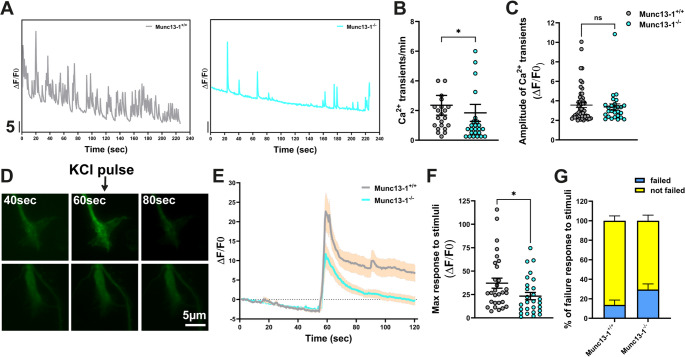
Neuronal activity is attenuated in Munc13-1 KO motoneurons.**A** and **B** Ca^2+^ imaging reveals decreased spontaneous Ca^2+^ transients in axonal growth cones of Munc13-1 KO motoneurons (*P = 0.0383, *n* = 22–25 cells, *N* = 4 independent cultures/mice for each genotype). **C** The amplitude of spontaneous Ca^2+^ transients is not altered in Munc13-1 KO motoneurons (*n* = 27–55 Ca^2+^ transients from 22–25 cells, *N* = 4 independent cultures/mice for each genotype). **D**-**F** The maximum response to a KCl depolarization pulse is diminished in axonal growth cones of Munc13-1 KO motoneurons (*P = 0.0385, *n* = 27–29 cells, *N* = 4 independent cultures/mice for each genotype). **G** Graph indicates the percentage of failure response to membrane depolarization in control and Munc13-1 KO motoneurons (*N* = 4 independent cultures/mice for each genotype). Data are presented as mean ± SEM. *P ≤ 0.05 (Two-tailed Mann-Whitney U test)

Intriguingly, these abnormalities in AZ assembly and VGCC clustering correlated with impaired neuronal activity in Munc13-1 KO motoneurons. As demonstrated by Ca^2+^ imaging, Munc13-1 KO motoneurons exhibit reduced spontaneous Ca^2+^ transients in growth cones (Fig. 5A, B), although the amplitude of these transients was not altered (Fig. 5C). Additionally, we applied a KCl depolarization to measure the evoked Ca^2+^ transient response (Fig. 5D). The maximum response to induced depolarization was diminished in growth cones of Munc13-1 KO motoneurons (Fig. 5E, F), and the percentage of failed responses to membrane depolarization was higher (Fig. 5G). In summary, these findings indicate that depletion of Munc13-1 leads to defects in growth cone differentiation, reduced levels of AZ components, including the VGCCs, which together contribute to reduced neuronal activity in motoneurons.

### Munc13-2 synaptic functions are compensated by Munc13-1

**Fig. 6:**
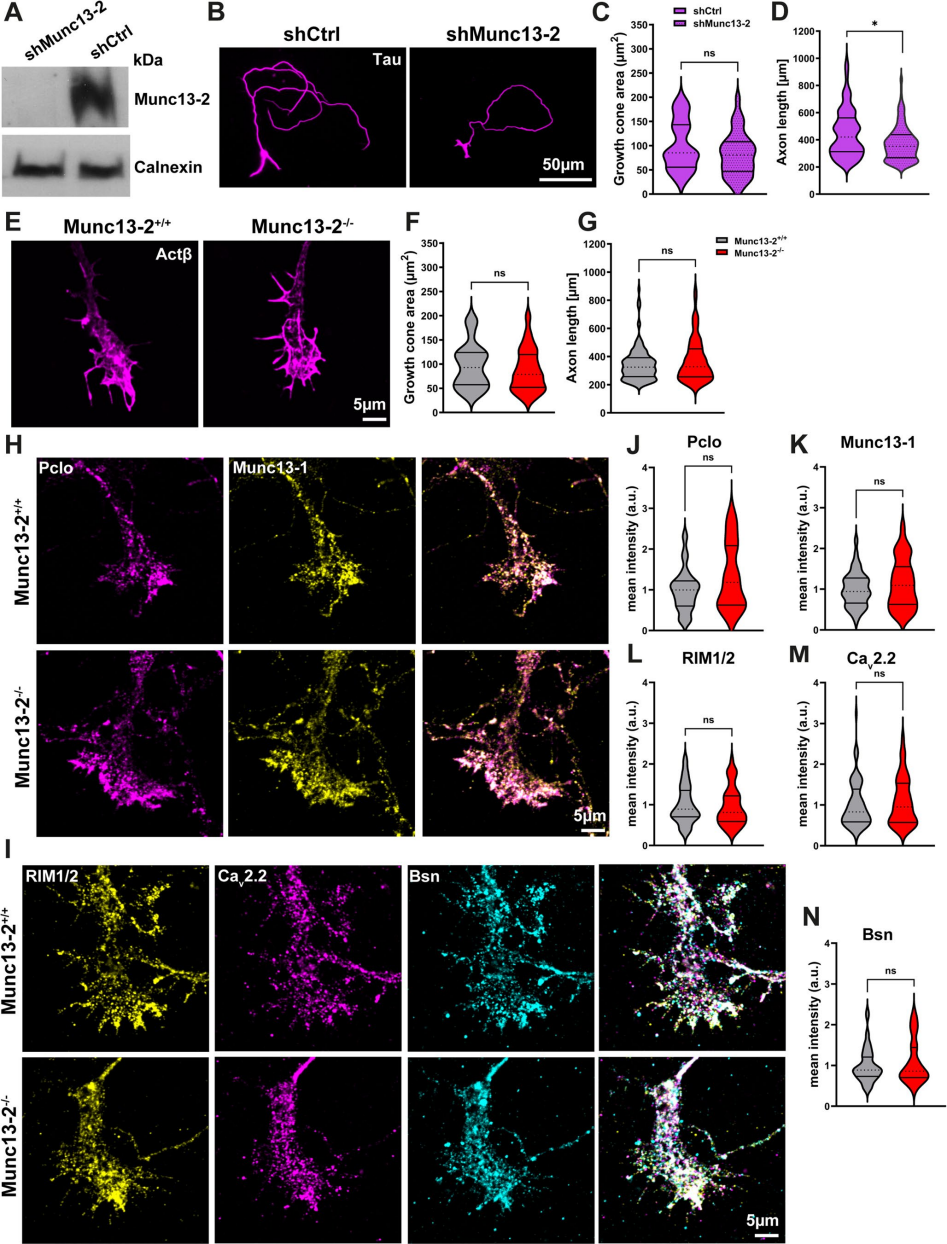
AZ assembly is not altered in Munc13-2 KO motoneurons.**A** Western blot with total lysates from cultured motoneurons reveals reduced Munc13-2 protein levels after lentivirus transduction with shMunc13-2. **B** Representative images of cultured shCtrl- and shMunc13-2-transduced motoneurons, stained against Tau. **C** and **D** Graphs show that Munc13-2 downregulation does not affect axonal growth cone area (*n* = 36–45 cells), but impairs axon outgrowth (**D**: *P = 0.05, *n* = 211–215 cells) in cultured motoneurons (*N* = 3 independent cultures/mice). **E** Representative images of axonal growth cones of control and Munc13-2 KO motoneurons stained against β-actin. **F** and **G** The area of axonal growth cones (*n* = 52–53 cells), as well as the axon length (*n* = 131–136 cells), are comparable between control and Munc13-2 KO motoneurons (*N* = 3 independent cultures/mice for each genotype). **H** and **I** Representative images of axonal growth cones of control and Munc13-2 KO motoneurons stained against AZ proteins. **J**-**N** Munc13-2 KO motoneurons exhibit unaltered levels of AZ components in axonal growth cones (*n* = 31–93 cells, *N* = 3 independent cultures/mice for each genotype). Data are presented as violin plots with the median shown as dashed lines. *P ≤ 0.05 (One-tailed Mann-Whitney U test)

To assess the role of Munc13-2 in motoneurons, we knocked down Munc13-2 using lentivirus transduction and analyzed its subsequent effects on AZ assembly and axonal growth and differentiation. Western blot analysis confirmed a reduction in Munc13-2 protein levels in motoneurons transduced with shRNA targeting Munc13-2 (Fig. 6A). Downregulation of Munc13-2 did not affect the area of axonal growth cones but impaired axon outgrowth in cultured motoneurons (Fig. 6B-D). Next, we analyzed the axon growth and differentiation in cultured Munc13-2 KO motoneurons. Our data revealed that the area of axonal growth cones and the axon length of Munc13-2 KO motoneurons were comparable to those of the control (Fig. 6E-G). Moreover, immunostaining of AZ proteins revealed unaltered levels of AZ components in axonal growth cones of cultured Munc13-2 KO motoneurons (Fig. 6H-N), suggesting that Munc13-2 is not essential for AZ assembly or growth cone differentiation in spinal motoneurons.

**Fig. 7:**
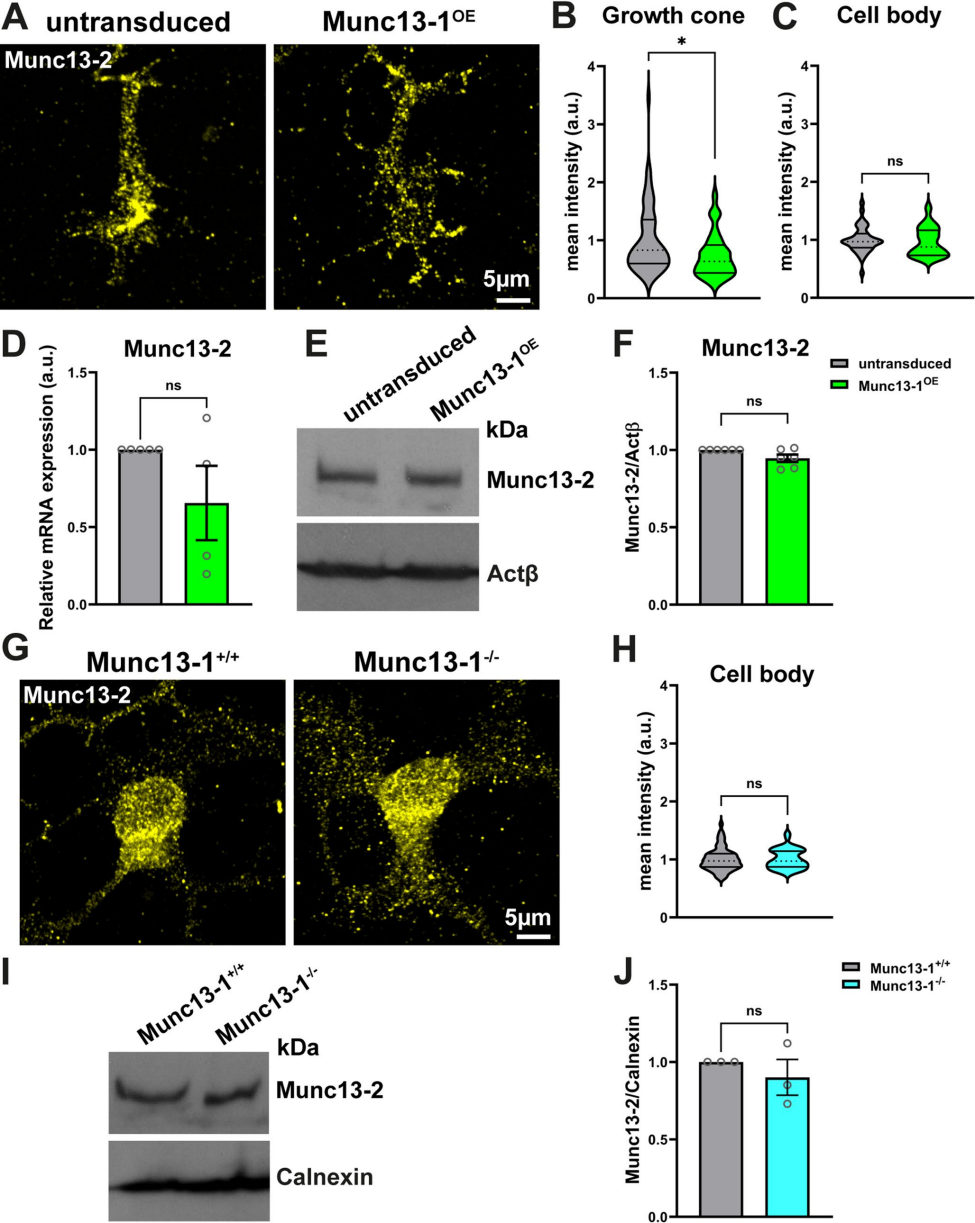
Loss of Munc13-2 synaptic functions is compensated by Munc13-1.**A** Representative images of axonal growth cones of motoneurons after transduction with a Munc13-1 overexpressing lentiviral construct (Munc13-1^OE^). **B** and **C** Graphs show a significant reduction in Munc13-2 levels in axonal growth cones (**B**: *P = 0.0143, *n* = 60–62 cells), but unaltered levels in cell bodies of Munc13-1^OE^-transduced motoneurons (**C**: *n* = 40 cells, *N* = 4 independent cultures/mice). **D** qRT-PCR shows unaltered mRNA levels of *Munc13-2* in total lysates from Munc13-1^OE^-transduced motoneurons (*N* = 4 independent cultures/mice). **E** and **F** Representative Western blot and quantification show unaltered levels of Munc13-2 in total lysates from Munc13-1^OE^-transduced motoneurons (*N* = 6 independent cultures/mice). **G** Representative images of cell bodies of Munc13-1 KO motoneurons stained against Munc13-2. **H** Graph shows unaltered Munc13-2 levels in cell bodies of Munc13-1 KO motoneurons (*n* = 62–63 cells, *N* = 3 independent cultures/mice for each genotype). **I** and **J** Representative Western blot and quantification show unaltered levels of Mnc13-2 in total lysates from Munc13-1 KO motoneurons (*N* = 3 independent cultures/mice for each genotype). In **B**, **C**, and **H**, data are presented as violin plots with the median shown as dashed lines. In **D**, **F** and **J** data are presented as mean ± SEM. *P ≤ 0.05 (One-tailed Mann-Whitney U test)

To explore potential compensatory mechanisms for Munc13-2 loss, we analyzed the effects of Munc13-1 overexpression in motoneurons. For this purpose, we generated a lentiviral construct expressing Munc13-1 (Munc13-1^OE^) and confirmed its overexpression in cultured motoneurons through immunostaining and Western blot assays (Fig. S4A-C). Immunostaining revealed that upon transduction of motoneurons with Munc13-1^OE^ virus, Munc13-2 levels decreased in axonal growth cones, while its levels remained unaltered in the soma (Fig. 7A-C). To further verify that Munc13-1 overexpression does not alter the total expression of Munc13-2, we conducted qRT-PCR and Western blot. The obtained data revealed no significant changes in *Munc13-2* mRNA levels in total lysates from Munc13-1^OE^-transduced motoneurons (Fig. 7D). This indicates that the specific reduction in Munc13-2 protein in axonal growth cones was not due to its transcriptional downregulation. Western blot analysis further confirmed that overall protein levels of Munc13-2 remain unaltered following Munc13-1 overexpression (Fig. 7E, F). Interestingly, unlike Munc13-2, overexpression of Munc13-1 in motoneurons led to increased levels of Ca_v_2.2 in axonal growth cones (Fig. S4D) without affecting the overall levels of other AZ proteins (Fig. S4E-H). These data unravel a specific and non-redundant role for Munc13-1 in VGCC tethering.

To determine whether a similar compensatory mechanism occurs for Munc13-1 loss through Munc13-2, we measured the Munc13-2 levels in cultured Munc13-1 KO motoneurons. Notably, we observed that Munc13-2 levels remained unchanged in axonal growth cones of Munc13-1 KO motoneurons (Fig. 4H, J). Consistent with this finding, total Munc13-2 levels were not elevated in Munc13-1 KO motoneurons, as demonstrated by immunostaining of cell bodies and Western blot analysis (Fig. 7G-J).

In conclusion, while Munc13-2 downregulation affects axon growth, it does not disrupt AZ assembly or growth cone differentiation. Furthermore, these data suggest that Munc13-1 has redundant functions with Munc13-2 and can compensate for Munc13-2 in motoneurons, thereby maintaining synaptic functions despite the loss of Munc13-2.

## Discussion

The present study provides new insights into the role of Munc13 proteins in the pathogenesis of synaptic dysfunctions in SMA. Our findings reveal that Munc13-1, but not Munc13-2, plays a critical role in the organization of AZs and the clustering of VGCCs in motoneurons, suggesting that Munc13-1 dysregulation contributes to the synaptic defects observed in SMA.

A common therapeutic strategy for SMA involves increasing SMN protein levels through gene therapy approaches, referred to as SMN-based therapies. These treatments target the *SMN2* gene to enhance SMN protein production [32]. However, non-responsive cases often arise due to two main factors: patients who miss the therapeutic window benefit less from SMN-repletion therapies, and there is still a significant knowledge gap in understanding how SMN deficiency leads to NMJ dysfunction and synapse degeneration. Understanding the cellular mechanisms driving synaptic deficiencies is therefore critical for identifying disease modifiers and developing combination therapies to improve patient outcomes. Our findings indicate that Munc13-1 depletion in motoneurons results in reduced levels of presynaptic AZ proteins and impairs the functionality of VGCCs in axonal growth cones, leading to reduced neuronal responsiveness. This is consistent with previous studies highlighting the Munc13-1 ability to tether VGCCs to SVs [14, 33], and defective neurotransmitter release in its absence in hippocampal neurons and at neuromuscular junctions [16, 18]. In line with this, we observed reduced Munc13-1 levels in presynaptic terminals of cultured Smn KO motoneurons and at NMJs in Smn KO mice. These findings provide a molecular mechanism that links Smn deficiency to synaptic abnormalities in SMA. Our results are consistent with previous findings demonstrating that UNC13A plays a critical role in ALS and frontotemporal dementia [34–36], where genetic variants together with TDP-43 loss of function lead to aberrant splicing, including the insertion of a cryptic exon [37, 38]. This mis-splicing reduces UNC13A function and compromises synaptic integrity. These observations further support the presence of overlapping molecular mechanisms between ALS and SMA.

Interestingly, although Munc13-2 also localizes to axonal growth cones, its role appears to be less crucial in motoneurons. Our data show that the loss of Munc13-2 does not significantly affect AZ assembly or growth cone differentiation, suggesting that Munc13-1 can compensate for the absence of Munc13-2. Conversely, in Munc13-1 KO motoneurons, the Munc13-2 levels remained unchanged, suggesting that Munc13-1 cannot be fully compensated by Munc13-2 in motoneurons. This lack of compensation may contribute to the synaptic deficits observed in SMA, emphasizing the non-redundant function of Munc13-1 in maintaining synaptic function. Moreover, the failure to upregulate Munc13-2 in response to Munc13-1 loss suggests that therapeutic strategies aimed at increasing Munc13-2 expression may not be sufficient to restore synaptic function in SMA. Nevertheless, in this study, we did not directly test the reciprocal rescue capacity of each isoform in the other’s loss-of-function background. Future studies using genetic models allowing for controlled isoform-specific expression in defined knockout backgrounds will be essential to fully elucidate the extent and directionality of functional compensation between Munc13 isoforms.

In neurons, subcellular mRNA localization and intra-axonal local translation are conserved cellular mechanisms that regulate synaptogenesis, plasticity, and axon regeneration through temporal and spatial modulation of the local proteome [39–41]. Disruption in local translation impacts neuronal function and viability, leading to neurodegeneration in ALS, SMA, Fragile X Syndrome, Alzheimer’s disease, and Parkinson’s disease [39, 42, 43]. Local translation is particularly crucial for spinal motoneurons due to their long axons and their unique anatomical and morphological characteristics [44]. These distinct features make motoneurons especially vulnerable to degeneration resulting from disruptions in protein synthesis, particularly within the axon. Impaired local translation in SMA is attributed to disrupted mRNA subcellular localization [29, 45], and the loss of direct interaction between the SMN protein and ribosomes, which is essential for the recruitment of mRNA onto polysomes [46]. Moreover, loss of SMN is associated with disturbed ribosome assembly and impaired formation of the rough endoplasmic reticulum (RER) in presynaptic compartments in motoneurons, leading to delayed translation initiation [31]. Here, we demonstrate that Smn downregulation results in a significant reduction in *Munc13-1* and *Liprinα1* mRNAs in axons of primary cultured mouse motoneurons. This finding highlights the importance of mRNA transport and local translation of synaptic proteins for the maintenance of synaptic integrity. This aligns with previous reports that Smn plays a critical role in mRNA localization and that its deficiency leads to widespread defects in axonal protein synthesis [7, 8, 47]. The mechanisms by which Smn regulates the axonal translocation of *Munc13-1* mRNA in motoneurons may involve the RNA-binding protein heterogeneous nuclear ribonucleoprotein R (hnRNP R). Importantly, iCLIP assays have revealed that *Munc13-1* mRNA binds to hnRNP R via its 3’ untranslated region (3’UTR) [48]. This interaction suggests that the correct axonal localization of Munc13-1 depends on the presence of hnRNP R within axons. hnRNP R is a binding partner of Smn, and in Smn KO motoneurons, axonal levels of hnRNP R are reduced [49]. Consequently, reduced levels of hnRNP R in axons of Smn KO motoneurons may disrupt the localization of *Munc13-1* mRNAs within axons, potentially affecting axonal function. Nevertheless, to confirm this mechanism, additional rescue experiments involving hnRNP R overexpression in Smn KO motoneurons will be required.

Thus, to fully restore synaptic function in SMA, therapies targeting SMN function may need to focus as well on correcting the mRNA transport and local translation processes. In conclusion, our findings demonstrate that Munc13-1 is pivotal for AZ assembly and neurotransmitter release in motoneurons, and its dysregulation contributes to the synaptic defects observed in SMA. The inability of Munc13-2 to fully compensate for Munc13-1 loss underscores the non-redundant functions of these proteins in maintaining synaptic integrity. These insights provide a deeper understanding of the molecular mechanisms underlying synaptic dysfunction in SMA and may inform the development of targeted therapies aimed at mitigating synaptic loss in this devastating disease.

## Supplementary Information

Below is the link to the electronic supplementary material.

Supplementary Material 1

## Data Availability

All datasets are provided within the main text or Supplementary Materials and can be obtained from the corresponding authors upon request. This study includes no data deposited in external repositories.
