# Supplementary material for: Loss of synaptic Munc13-1 underlies neurotransmission abnormalities in spinal muscular atrophy
Source: Cell Mol Life Sci. 2025 Aug 29;82(1):325. doi: 10.1007/s00018-025-05859-7 (PMC12397458; doi:10.1007/s00018-025-05859-7)
Supplement: Supplementary file 1 — Supplementary Material 1 [file 18_2025_5859_MOESM1_ESM.docx]

**Supplementary Data**

**
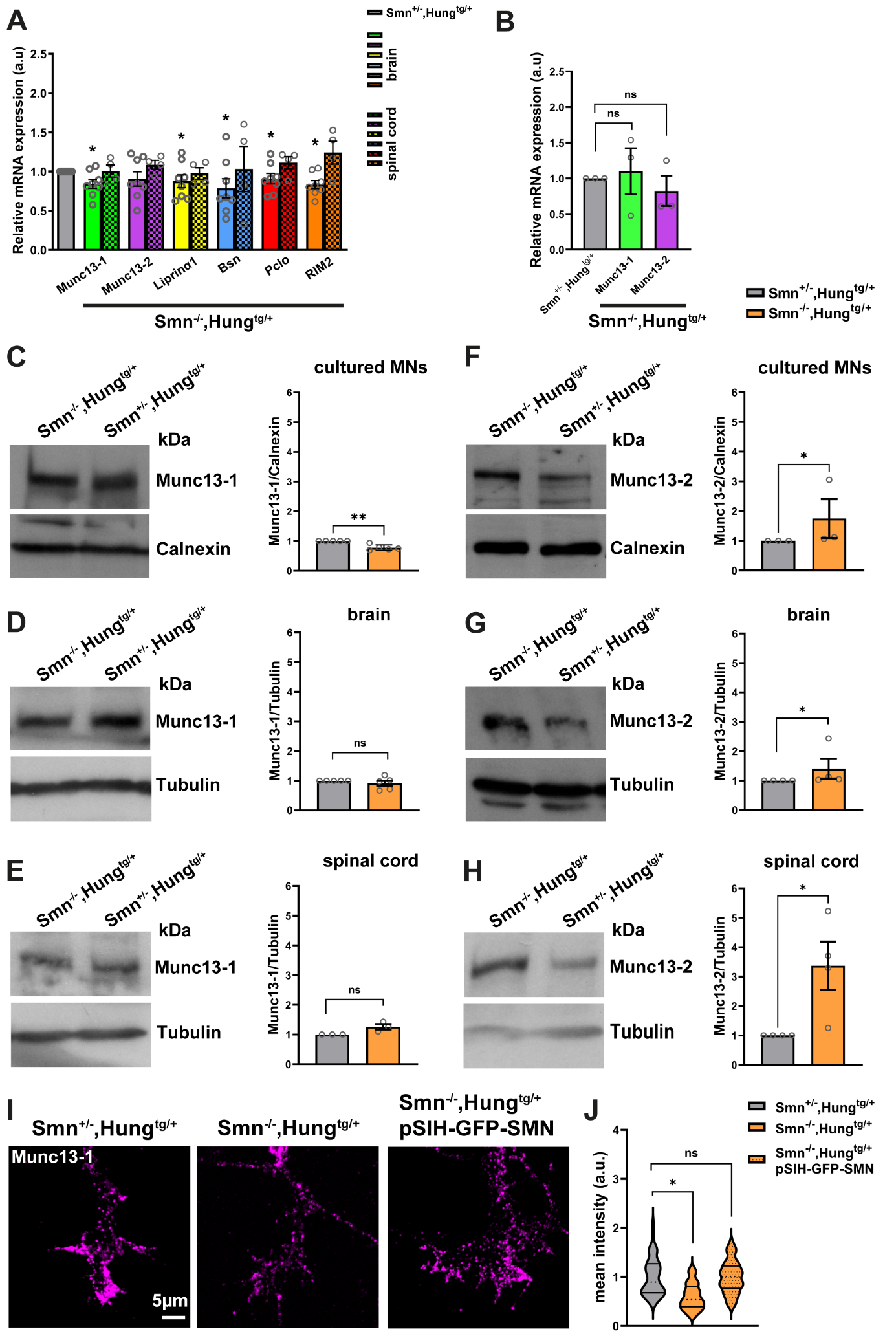
**

**Fig. S1 Expression of Munc13 isoforms in brain and spinal cord tissues from Smn KO mice.**

**A** qRT-PCR was used to assess the total mRNA expression levels of AZ components in whole-tissue lysates from the brain and spinal cord of P10 control and Smn KO mice. In brain lysates from Smn KO mice, qRT-PCR analysis revealed a significant reduction in the expression of several AZ transcripts, including *Munc13-1*, *Liprinα1*, *Bassoon* (Bsn), *Piccolo* (Pclo), and *RIM* (*P = 0.0454, N = 8 independent experiments), indicating that Smn loss impairs the expression of multiple presynaptic mRNAs in the brain. Notably, *Munc13-2* mRNA levels were not significantly changed, suggesting isoform-specific regulation. In contrast, mRNA expression levels of these synaptic components were not significantly altered in whole-lysates from spinal cords from Smn KO mice compared to controls (N = 4 independent experiments). **B** qRT-PCR shows mRNA expression of Munc13s in cultured control and Smn KO motoneurons (N = 3 independent cultures/mice for each genotype). **C**-**H** Representative immunoblots and corresponding quantifications show Munc13s expression in total lysates from cultured motoneurons (**C**: **P = 0.0039, N = 5 independent cultures/mice for each genotype, **F**: *P = 0.05, N = 3 independent cultures/mice for each genotype), brain (**D**: N = 5 independent experiments, **G**: *P = 0.0143, N = 4 independent experiments), and spinal cord (**E**: N = 3 independent experiments, **H**: *P = 0.0276, N = 4 independent experiments) tissues of Smn KO mice. (**I**) Representative images of axonal growth cones of cultured motoneurons immunostained for Munc13-1. (**J**) Quantification of the immunosignal, as represented in panel **I**, reveals increased Munc13-1 protein levels in axonal growth cones of Smn KO motoneurons compared to Smn KO (*P = 0.05, n = 59-63 cells, N = 3 independent cultures/mice for each genotype). In **A**-**H**, data are presented as mean ± SEM. In **J**, data are presented as violin plot with the median shown as dashed lines. *P ≤ 0.05 (One-tailed Mann-Whitney U test).


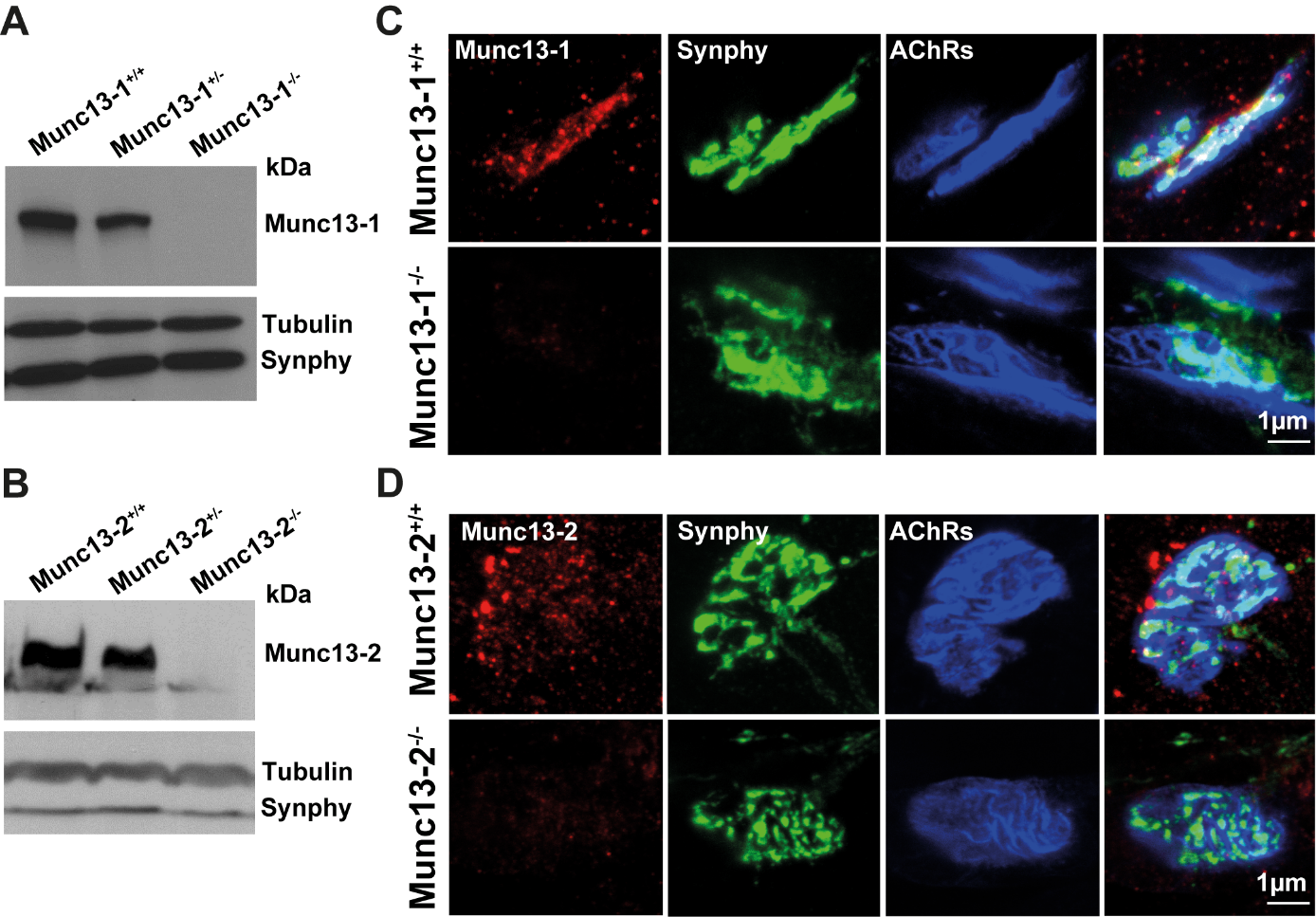


**Fig. S2 Validation of Munc13-1 and Munc13-2 antibody specificity using Munc13s KO mice.**

**A** and **B** Representative immunoblots from crude synaptosome fractions obtained from cortical tissues of Munc13-1 and Munc13-2 KO mice show the specificity of implied Munc13-1 and Munc13-2 antibodies. **C** and **D** Representative images of NMJs from the diaphragm of P0 Munc13-1, and P5 Munc13-2 KO mice showing antibody specificity.

**
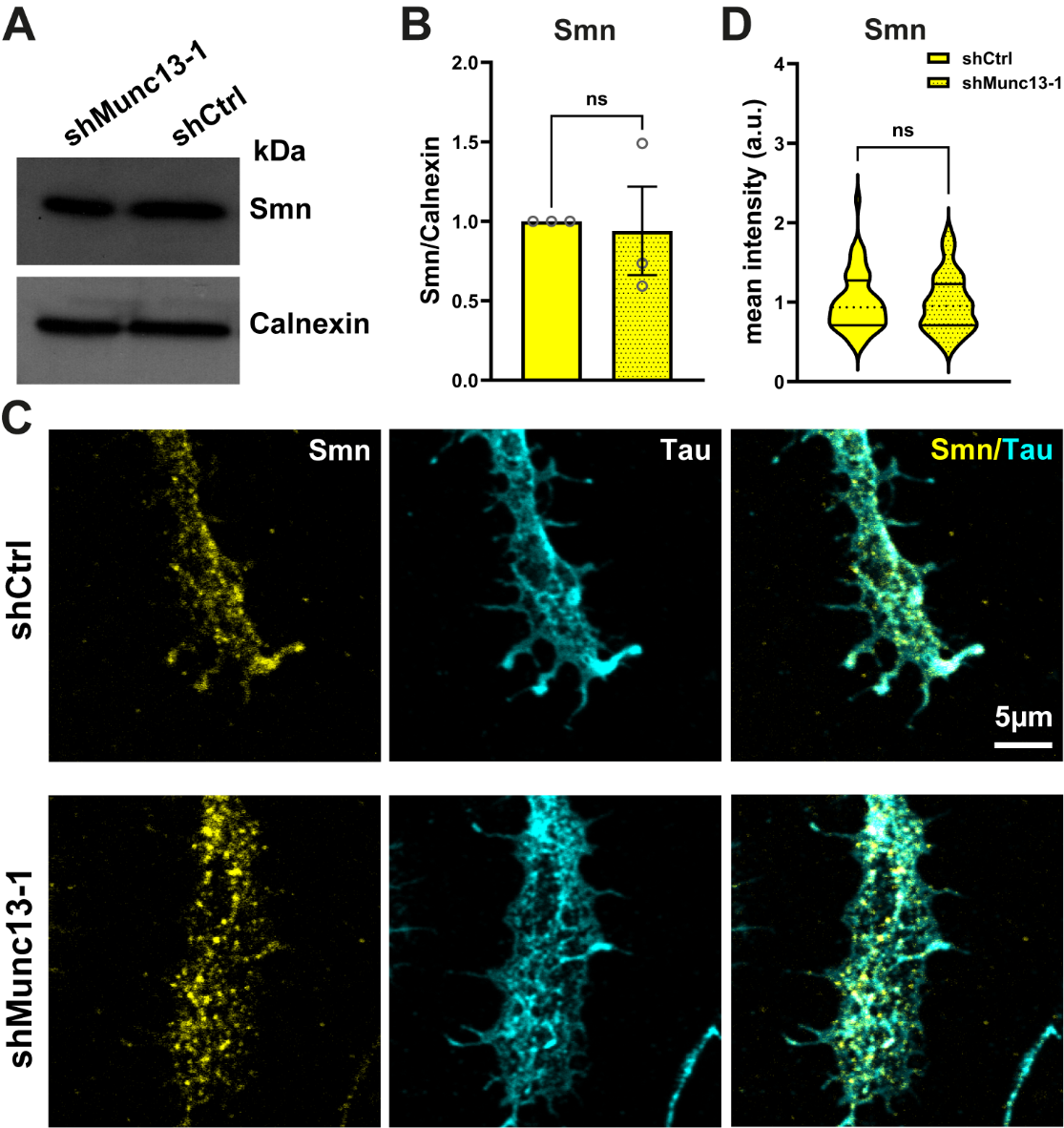
**

**Fig. S3 Munc13-1 is not required for Smn expression.**

**A** Representative immunoblot of total lysates from cultured control and Munc13-1 knockdown motoneurons, indicating Smn levels. Calnexin served as loading control. **B** Quantification of the Western blots shows that Smn protein levels remain unchanged following Munc13-1 knockdown (N = 3 independent cultures/mice). **C** Representative images of axonal growth cones of cultured control and Munc13-1 knockdown motoneurons immunostained against Smn. **D** Quantification of the immunosignal, as represented in panel **C**, reveals that shRNA-mediated Munc13-1 depletion does not affect Smn protein levels in axonal growth cones of cultured motoneurons (n = 50-56 cells, N = 3 independent cultures/mice). In **B**, data are presented as mean ± SEM. In **D**, data are presented as violin plot with the median shown as dashed lines. *P ≤ 0.05 (One-tailed Mann-Whitney U test).


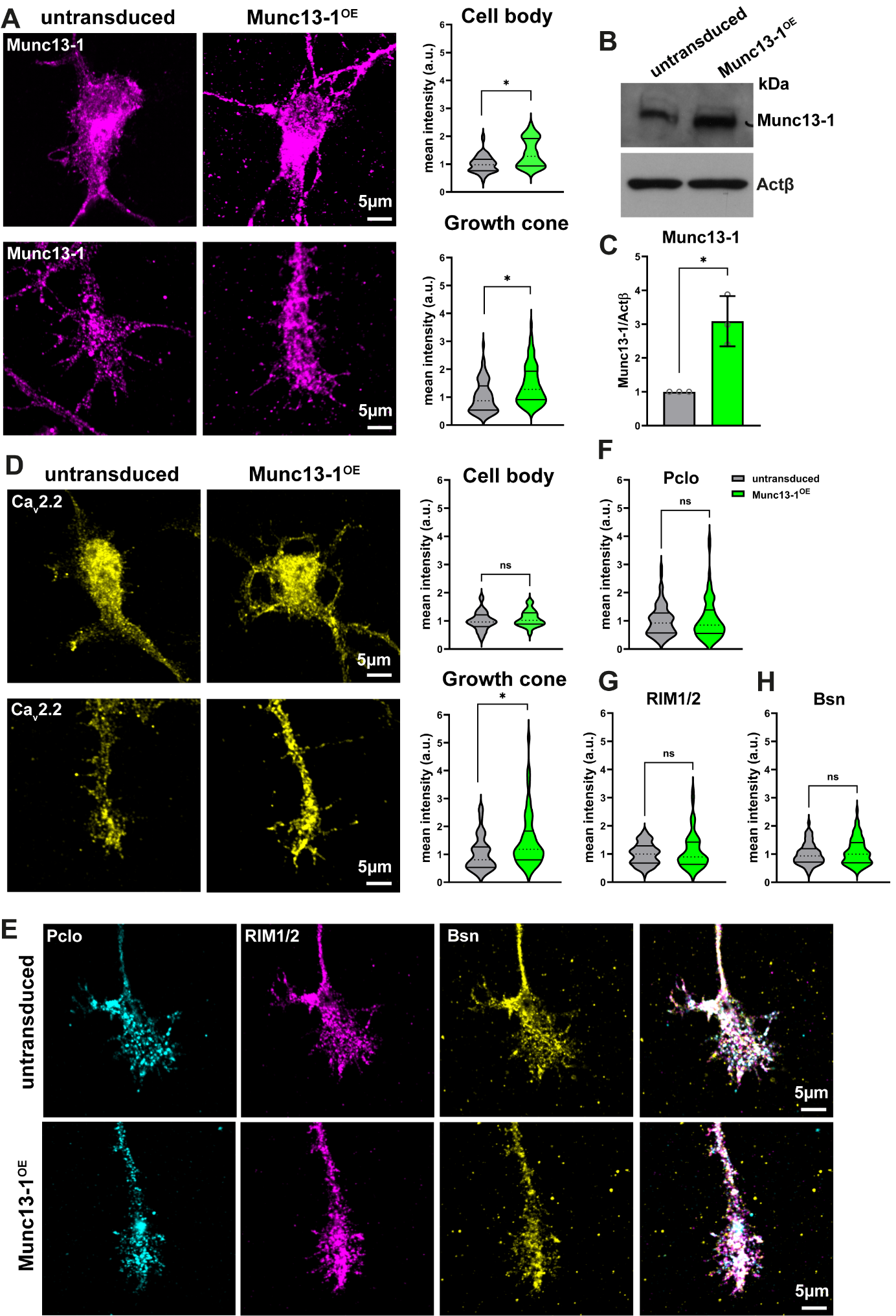


**Fig. S4 Validation of the Munc13-1 overexpressing lentivirus construct.**

**A** Representative images of cell bodies and axonal growth cones of cultured motoneurons and their corresponding quantifications indicate increased Munc13-1 levels in cultured motoneurons transduced with Munc13-1 overexpressing lentivirus (*P = 0.05, n = 58-62 cell bodies, and n = 79-82 axonal growth cones, N = 3 independent cultures/mice). **B** and **C** Representative immunoblot and the corresponding quantification (**C**: *P = 0.05, N = 3 independent cultures/mice) show upregulation of Munc13-1 upon lentivirus transduction with Munc13-1^OE^ construct. **D** Representative images of cultured motoneurons and their corresponding quantifications show increased Ca_v_2.2 levels in axonal growth cones of Munc13-1^OE^-transduced motoneurons (**P = 0.0058, n = 42-57 axonal growth cones, and P = 0.499, n = 19-23 cell bodies, N = 3 independent cultures/mice). **E** Representative images of axonal growth cones of cultured Munc13-1^OE^-transduced motoneurons, stained against AZ components. **F**-**H** Graphs show comparable levels of Pclo (n = 75-84 cells), RIM1/2 (n = 75-84 cells), and Bsn (n = 69-82 cells) in Munc13-1^OE^-transduced motoneurons compared to control (N = 4 independent cultures/mice). In **A**, **D** and **F**-**H**, data are presented as violin plots with the median shown as dashed lines. In **C** data are presented as mean ± SEM. *P ≤ 0.05 (One-tailed Mann-Whitney U test).
